# Outcomes in Registered, Ongoing Randomized Controlled Trials of Patient Education

**DOI:** 10.1371/journal.pone.0042934

**Published:** 2012-08-16

**Authors:** Cécile Pino, Isabelle Boutron, Philippe Ravaud

**Affiliations:** 1 INSERM, U738, AP-HP (Assistance Publique des Hôpitaux de Paris), Hôpital Hôtel Dieu, Centre d’Epidémiologie Clinique, Paris, France; 2 Université Paris Descartes, Faculté de Médecine, Paris, France; 3 Centre Cochrane Français (EHESP, HAS, INSERM, APHP), Paris, France; Universidad Peruana Cayetano Heredia, Peru

## Abstract

**Background:**

With the increasing prevalence of chronic noncommunicable diseases, patient education is becoming important to strengthen disease prevention and control. We aimed to systematically determine the extent to which registered, ongoing randomized controlled trials (RCTs) evaluated an educational intervention focus on patient-important outcomes (i.e., outcomes measuring patient health status and quality of life).

**Methods:**

On May 6, 2009, we searched for all ongoing RCTs registered in the World Health Organization International Clinical Trials Registry platform. We used a standardized data extraction form to collect data and determined whether the outcomes assessed were 1) patient-important outcomes such as clinical events, functional status, pain, or quality of life or 2) surrogate outcomes, such as biological outcome, treatment adherence, or patient knowledge.

**Principal Findings:**

We selected 268 of the 642 potentially eligible studies and assessed a random sample of 150. Patient-important outcomes represented 54% (178 of 333) of all primary outcomes and 46% (286 of 623) of all secondary outcomes. Overall, 69% of trials (104 of 150) used at least one patient-important outcome as a primary outcome and 66% (99 of 150) as a secondary outcome. Finally, for 31% of trials (46 of 150), primary outcomes were only surrogate outcomes. The results varied by medical area. In neuropsychiatric disorders, patient important outcomes represented 84% (51 of 61) of primary outcomes, as compared with 54% (32 of 59) in malignant neoplasm and 18% (4 of 22) in diabetes mellitus trials.

In addition, only 35% assessed the long-term impact of interventions (i.e., >6 months).

**Conclusions:**

There is a need to improve the relevance of outcomes and to assess the long term impact of educational interventions in RCTs.

## Introduction

Over the past 2 decades, the World Health Organization (WHO) has pointed to the increasing prevalence of chronic diseases and has called for a reorientation of health systems to efficiently strengthen disease prevention and control[1]. The proportion of deaths due to noncommunicable diseases globally is expected to increase from 59% in 2002 to 69% in 2030[1].

Patient education through various educational interventions should play an essential role for adequate understanding and managing chronic diseases[2], such as non-communicable or infectious diseases. In fact, interventions designed to improve treatment compliance or strengthen behaviour changes are as important as effective pharmacological treatments in reducing the burden of chronic diseases.

Finding ways to support patients with chronic illness is an important focus in the development of health care agendas. In 2009, the American Institute of Medicine developed a priority list of 100 research topics that should be addressed by comparative effectiveness research[3]; educational interventions represented about 30% of the topics in the first quartile of the priorities. To meet these requirements, we need educational interventions that have demonstrated their effectiveness for relevant outcomes.

Our aim was to assess the relevance of the upcoming evidence in the field of patient education. We systematically determined the extent to which registered, ongoing randomized controlled trials (RCTs) evaluating an educational intervention focus on patient-important outcomes.

## Methods

### Search strategy

The sample of RCTs assessed in the study was used and described in a previous work[4]. In brief, on May 6, 2009, we searched for all ongoing RCTs assessing an educational intervention registered in the 10 registries accessible by the WHO search portal[5]: Australian New Zealand Clinical Trials Registry, Chinese Clinical Trial Register, ClinicalTrials.gov, Clinical Trials Registry – India, German Clinical Trials Register, Iranian Registry of Clinical Trials, ISRCTN.org, Sri Lanka Clinical Trials Registry, The Netherlands National Trial Register, EU Clinical Trials Register. We used this platform because it allows for access to all primary registries meeting the WHO criteria. We used the key word “education” in the field “intervention” and the filter “recruiting” in the field “recruitment status” in the “advanced search” of the WHO search portal.

### Study selection

Eligibility criteria were ongoing study (recorded as “recruiting” in the field “recruitment status”), RCT (recorded as “randomised” in the field “study design” or described as randomised in the description of the intervention), evaluating an educational intervention (i.e., intervention designed to teach or train patients concerning their own health[2]) and dedicated to participants (healthy or sick), their family members and home caregivers. We excluded interventions involving health workers if the intervention was integrally delivered to them, as well as curative interventions designed to treat mental disorders, such as psychotherapy interventions.

Two reviewers independently screened all potentially eligible studies. All disagreements were resolved by consensus and with a third reviewer if necessary.

From the selected panel, we randomly selected a sample of 150 studies for assessment.

### Data collection

A data extraction form was developed and tested beforehand on a sample of 20 studies by 2 independent reviewers. The agreement rate ranged from p = 0.7 to 1.

A single reviewer used the extraction form to collect data 1) from the record available in the WHO International Clinical Trials Registry Platform, 2) from the record available in the primary registry, and 3) from a website referenced in the record or from previous publications if listed in the record. We did not perform any additional Internet search if no reference was mentioned in the record.

To classify outcomes, the following procedure was used: 1) one of the authors extracted all outcomes reported in the registries; 2) all 3 authors classified these outcomes into 2 categories (patient-important or surrogate outcomes), according to prior works on this topic[2], [6], [7], [8], [9], [10], [11]. All disagreements were discussed until consensus was reached.

### Study design and general characteristics

We recorded the name of the primary registry, date of trial registration, study design, medical area, experimental intervention, comparator, sample size, number of arms and number of experimental arms. We also recorded the blinding characteristics, randomization process and population of analysis.

### Type of outcomes

Our classification was developed according to previous works on this topic conducted in various medical domains [2], [6], [7], [8], [9], [10], [11] and distinguished 2 groups: 1) patient-important outcomes and 2) surrogate outcomes.

We defined a patient-important outcome as “a characteristic or variable that reflects how a patient feels, functions, or survives”[12], that is, all outcomes leading to important changes for patient life. These were for example clinical events, functional status, pain and quality of life. Surrogate outcomes were defined as “a characteristic intended to substitute for a clinical endpoint”[12], [13]. These were typically biochemical markers, physiological parameters or subclinical endpoints that were not generally perceived directly by patients, but were nevertheless associated with patient-important outcomes[14]. For example, high blood pressure does not reflect how a patient feels, functions, or survives but is known to be associated with increased risk of stroke. Other surrogate outcomes were for example treatment adherence, patient knowledge, satisfaction and acceptability of experimental interventions.

Finally, we checked whether at least one long-term outcome (>6 months) was considered in the study, as is widely recommended ^11^.

### Statistical analysis

We computed descriptive statistics for continuous variables: median, minimum and maximum values. Categorical variables were described with frequencies and percentages. All data analyses involved use of R for Windows, Release 2. 9.

## Results

### Studies selected

Figure S1 describes the selection of trial records. We screened 642 trials for eligibility and 268 finally met our inclusion criteria.

From the selected panel, we randomly selected a sample of 150 studies for assessment.

### General characteristics and study design

All selected trials were registered in 4 registers, mainly ClinicalTrials.gov (61%) (Table S1). The median [IQR] of the targeted sample size was 205 [100–400]. The comparator(s) were mainly usual care (47%) or active treatment (49%). Of these, most concerned other educational interventions.

In total, 70 trials (47%) were taking place in the United States and 38 (25%) in Europe; no study involved low-income economy countries. For trials reporting centre, most (64%) involved only one centre. Participating centres were mainly academic or university hospitals (51%) [4].

The experimental intervention was mainly (87%) programmed interaction, that is, individual or group educational sessions or phone calls; 30% evaluated the provision of educational material such as a DVD or booklet and 12% an Internet program [4].

Data relating to design characteristics were lacking, particularly the randomisation process (random sequence generation, 14%; allocation concealment, 13%), and the population of analysis (3%). Data on blinding were lacking or unclear in about one-quarter of the records. For trials with labelling information, most were open label (49%), and about 20% of the records reported a blinded outcome assessor.

In total, 132 trials focused on disease management and 28, a risk factor. The 3 most frequent diseases targeted by educational interventions were neuropsychiatric disorders, studied in 28 trials (21%), cardiovascular diseases in 27 (21%) and malignant neoplasm in 23 (17%) (Table S1).The principal risk factors studied were overweight (n = 11) and tobacco consumption (n = 6).

### Outcomes measured

For the 150 records, 148 (99%) reported primary outcome(s) and 132 (88%) secondary outcomes (Table S2). The metric used to measure outcomes was given for 104 trials (70%) and the time point for 123 (84%).

The total number of reported primary outcomes was 333, with a median [IQR] per trial of 1 [1–3; range 1–13].The total number of secondary outcomes was 623, with a median per trial of 3 [2–6; range 1–20].

Of 150 trials, 52 (34.7%) reported at least one long-term outcome.

Patient-important outcomes represented about 54% (n = 178) of all primary outcomes assessed; 47% (n = 157) assessed functional status, pain or quality of life and 7% (n = 21) clinical events.

Surrogate outcomes represented 44% (n = 141) of all primary outcomes assessed. Overall, 69% of trials (n = 104) used at least one patient-important outcome as a primary outcome and 66% (n = 99) as a secondary outcome. Finally, for 31% of trials (n = 46), primary outcomes were only surrogate outcomes.

The results varied by medical area. For neuropsychiatric disorders, patient important outcomes represented 84% (51 of 61) of primary outcomes, as compared with 54% (32 of 59) in malignant neoplasm and 18% (4 of 22) in diabetes mellitus trials.

## Discussion

### Principal findings

This study assessed a representative random sample of 150 ongoing RCTs assessing an educational intervention that were registered in the WHO database of trials. The reporting of methodological characteristics was lacking for most records, and about 9% of all outcomes assessed were unclear or were not reported. The interventions were mainly for neuropsychiatric disorders, cardiovascular diseases and malignant neoplasm. Patient-important outcomes represented 54% (n = 178) of all the primary outcomes assessed and surrogate outcomes 44% (n = 141). About 70% of the studies used at least one patient-important outcome as primary outcome, but one third used only a surrogate outcome. Furthermore, outcomes assessed varied by medical area. For neuropsychiatric disorders, patient important outcomes represented 84% (51 of 61) of primary outcomes, as compared with 54% (32 of 59) in malignant neoplasm and 18% (4 of 22) in diabetes mellitus trials.

Finally, only 35% of the selected trials reported assessing the long-term impact of interventions.

Our results are consistent with previous findings[6], [15], [16], [17], [18] and raise some issues. First, the use of surrogate outcomes is particularly problematic in studies of educational interventions, which showed a poor association of surrogate and relevant outcomes. Indeed, improving knowledge and coping skills does not necessarily lead to positive behavioural change, and such change does not always result in better health outcomes[2], [7], [8], [19].We found that one third of RCTs of educational interventions do not use patient-important outcomes, only surrogate outcomes, so some of the healthcare research conducted in patient education will be of little use for both clinicians and patients.

Second, our finding of few records reporting long-term follow-up highlights a lack of evaluating the sustained impact of interventions[20]. Long-term impact is an important issue[21] in patient education, because maintaining individual behavior change is particularly challenging.

Finally, our results highlight the poor reporting of important data such as methodological characteristics and outcomes in trial registration. The poor quality of registered information is problematic because it raises doubts about the ability of trial registration to achieve research transparency.

### Limitations

The inferences from this study are limited by the inherent limitations of trial registries. Our search strategy may not have identified all studies evaluating educational interventions. Registries have limited advanced search capabilities; contrary to search strategies for databases of published articles such as Medline or the Cochrane Central Register of Controlled Trials, search strategies for the WHO International Clinical Trials Registry platform have not been evaluated. In addition, all RCTs assessing an educational intervention may have not been registered or not fully registered in trial registries; however, whether the trial is registered or not probably does not depend on the relevance of outcomes assessed. Finally, our outcomes classification was developed according to previous works on this topic conducted in various medical fields [2], [6], [7], [8], [9], [10], [11], for example, in Ghandi's study[6].

## Conclusions

There is a need to improve the relevance of outcomes and to assess the long term impact of interventions in RCTs conducted in patient education field.

## Supporting Information

Figure S1 **Flow diagram of the selected studies.**
(TIFF)

Table S1 **General characteristics and study design of selected trials.**
(DOC)

Table S2 **Outcomes given in registration records for randomized controlled trials of educational interventions by primary or secondary outcome and medical area.**
(DOC)
