# Supplementary figures and images for: Outcomes in Registered, Ongoing Randomized Controlled Trials of Patient Education
Source: PLoS One. 2012 Aug 16;7(8):e42934. doi: 10.1371/journal.pone.0042934 (PMC3420885; doi:10.1371/journal.pone.0042934)

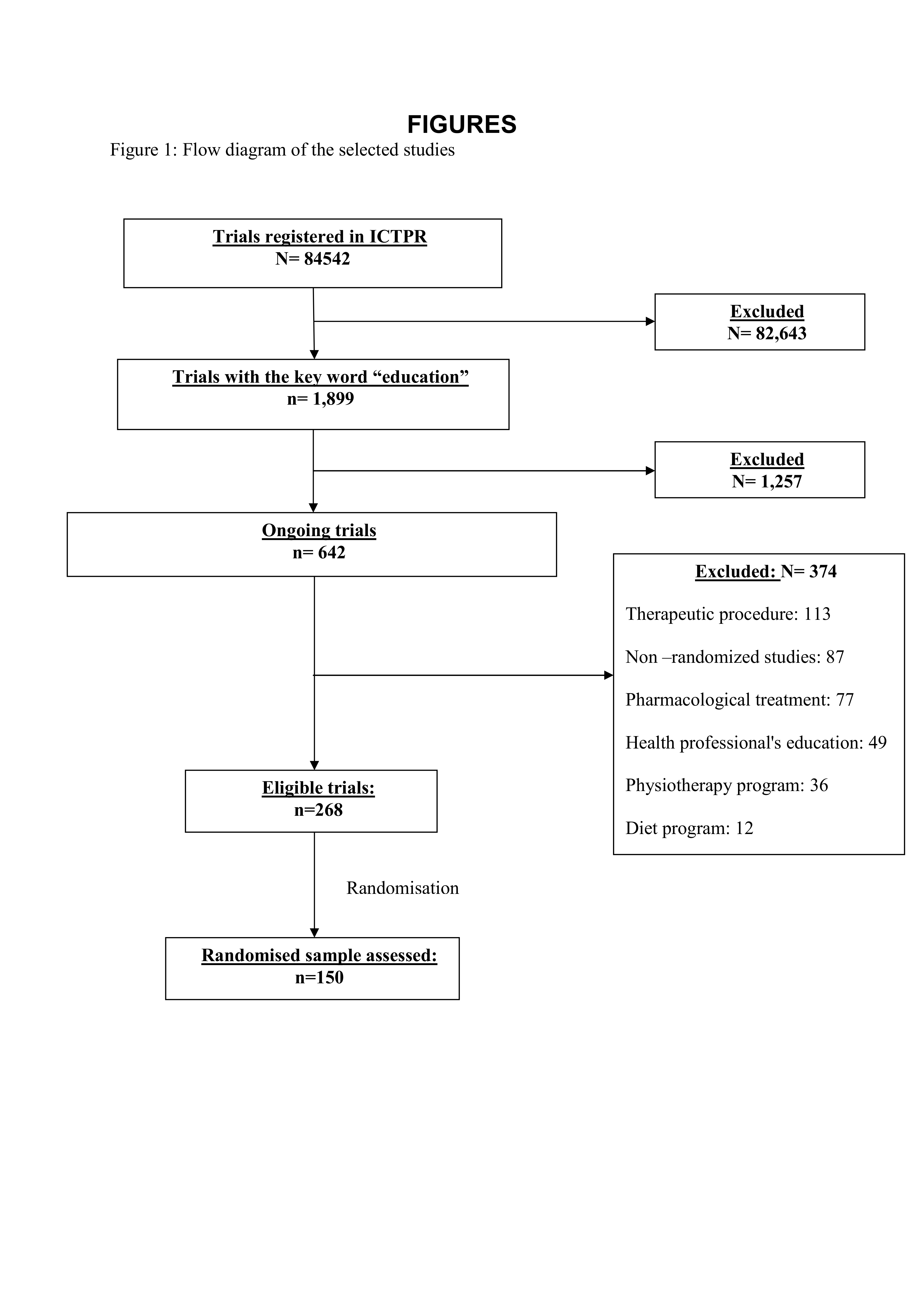

Supplement: Figure S1 — Flow diagram of the selected studies. (TIFF) [file pone.0042934.s001.tiff]
