# Supplementary material for: Outcomes in Registered, Ongoing Randomized Controlled Trials of Patient Education
Source: PLoS One. 2012 Aug 16;7(8):e42934. doi: 10.1371/journal.pone.0042934 (PMC3420885; doi:10.1371/journal.pone.0042934)
Supplement: Table S1 — General characteristics and study design of selected trials. (DOC) [file pone.0042934.s002.doc]

# Tables

Table S1: General characteristics and study design of selected trials

| **Items** | **N = 150**  n (%) |
| --- | --- |
| **Primary registry** |  |
| ClinicalTrials.gov | 91 (61) |
| ISRCTN.org | 31 (21) |
| Australian New Zealand Clinical Trials Registry | 18 (12) |
| The Netherlands National Trial | 10 (7) |
| **Date of Registration** |  |
| Before 2007 | 47 (31) |
| 2007–2009 | 103 (69) |
| **Study design** |  |
| Parallel groups | 132 (88) |
| Cross-over | 10 (7) |
| Factorial assignment | 8 (5) |
| **Target sample size, median [IQR]** | 205 [100–400] |
| **Medical area, n=132** |  |
| Neuropsychiatric disorders | 28 (21) |
| Cardiovascular disease | 27 (21) |
| Malignant neoplasm | 23 (17) |
| Musculoskeletal diseases | 10 (7) |
| Diabetes mellitus | 9 (7) |
| **Comparator[[1]](#footnote-2)** |  |
| Active treatment | 73 (49) |
| Drug | 2 (1) |
| Education | 70 (96) |
| Rehabilitation | 1 (1) |
| Usual care | 70 (47) |
| Placebo | 12 (8) |
| Unclear | 8 (5) |
| **“Blinding” characteristics, n=157** |  |
| Open label | 74 (49) |
| Evaluators blinded | 29 (19) |
| Subjects blinded | 12 (8) |
| Care providers blinded | 3 (2) |
| Unclear | 11 (7) |
| Not reported | 28 (19) |
| **Reporting of the sequence generation** | 21 (14) |
| **Reporting of the allocation concealment procedures** | 19 (13) |
| **Reporting of the population used for statistical analysis** | 5 (3) |

1. Multiple answers possible, so the total does not equal 100% [↑](#footnote-ref-2)
