# Supplementary material for: Outcomes in Registered, Ongoing Randomized Controlled Trials of Patient Education
Source: PLoS One. 2012 Aug 16;7(8):e42934. doi: 10.1371/journal.pone.0042934 (PMC3420885; doi:10.1371/journal.pone.0042934)
Supplement: Table S2 — Outcomes given in registration records for randomized controlled trials of educational interventions by primary or secondary outcome and medical area. (DOC) [file pone.0042934.s003.doc]

Table S2: Outcomes given in registration records for randomized controlled trials of educational interventions by primary or secondary outcome and medical area.

|  | **Patient-important outcomes** | **Surrogate outcomes** | **unclear or not reported** |
| --- | --- | --- | --- |
| **All outcomes** |  |  |  |
| Primary outcomes, n=333 | 178 (54) | 141 (44) | 14 (4) |
| Secondary outcomes, n=623 | 286 (46) | 321 (52) | 16 (3) |
| **Main medical areas** | |  |  |
| Neuropsychiatric disorders, n=61 | 51 (84) | 10 (16) | 0 |
| Cardiovascular disease, n=48 | 18 (38) | 29 (60) | 1 (2) |
| Malignant neoplasm, n=59 | 32 (54) | 24 (41) | 3 (5) |
| Musculoskeletal diseases, n=21 | 15 (71) | 5 (24) | 1 (5) |
| Diabetes mellitus, n=22 | 4 (18) | 18 (82) | 0 |
